# Evaluating the Performance of ChatGPT in the Prescribing Safety Assessment: Implications for Artificial Intelligence-Assisted Prescribing

**DOI:** 10.7759/cureus.73003

**Published:** 2024-11-04

**Authors:** David Bull, Dide Okaygoun

**Affiliations:** 1 Trauma and Orthopaedics, Chelsea and Westminster Hospital NHS Foundation Trust, London, GBR; 2 Intensive Care Unit, Barts Health NHS Trust, London, GBR

**Keywords:** artificial intelligence and education, artificial intelligence chatgpt-4, artificial intelligence in medicine, medical education assessment, medical student assessment, open book examination, prescribing safety, uk - united kingdom

## Abstract

Objective

With the rapid advancement of artificial intelligence (AI) technologies, models like Chat Generative Pre-Trained Transformer (ChatGPT) are increasingly being evaluated for their potential applications in healthcare. The Prescribing Safety Assessment (PSA) is a standardised test for junior physicians in the UK to evaluate prescribing competence. This study aims to assess ChatGPT's ability to pass the PSA and its performance across different exam sections.

Methodology

ChatGPT (version GPT-4) was tested on four official PSA practice papers, each containing 30 questions, in three independent trials per paper, with answers evaluated using official PSA mark schemes. Performance was measured by calculating overall percentage scores and comparing them to the pass marks provided for each practice paper. Subsection performance was also analysed to identify strengths and weaknesses.

Results

ChatGPT achieved mean scores of 257/300 (85.67%), 236/300 (78.67%), 199/300 (66.33%), and 233/300 (77.67%) across the four papers, consistently surpassing the pass marks where available. ChatGPT performed well in sections requiring factual recall, such as "Adverse Drug Reactions", scoring 63/72 (87.50%), and "Communicating Information", scoring 63/72 (88.89%). However, it struggled in "Data Interpretation", scoring 32/72 (44.44%), showing variability across trials and indicating limitations in handling more complex clinical reasoning tasks.

Conclusion

While ChatGPT demonstrated strong potential in passing the PSA and excelling in sections requiring factual knowledge, its limitations in data interpretation highlight the current gaps in AI’s ability to fully replicate human clinical judgement. ChatGPT shows promise in supporting safe prescribing, particularly in areas prone to human error, such as drug interactions and communicating correct information. However, due to its variability in more complex reasoning tasks, ChatGPT is not yet ready to replace human prescribers and should instead serve as a supplemental tool in clinical practice.

## Introduction

Integrating artificial intelligence (AI) into various sectors has ushered in a new era of technological advancement. The use of machines and systems to aid healthcare professionals is not new; in the 1970s, Mycin, a computer-based consultation system, was made to assist physicians in the selection of antibiotics for infections [1-3]. These systems required significant manual data entry, making it difficult to keep up-to-date with new advancements in antibiotics and medical knowledge [4,5]. The problems faced with old systems such as this are being demolished with new Large Language Models (LLM) such as Chat Generative Pre-Trained Transformer (ChatGPT), introduced by OpenAI (San Francisco, CA) in late 2022. These LLMs are a modern shift from rule-based systems, such as Mycin, to a generative model, which has the ability to learn from vast datasets and perform natural processing tasks that older systems couldn’t. Perhaps the most famous LLM, ChatGPT, is a simple-to-use chatbot with an interface similar to a messaging system, whereby the user inputs what is known as a prompt, and then ChatGPT will give a response [6]. Some responses that can be received from the system are impressive, as will be discussed below; however, whilst seemingly capable of performing high-level thinking in many contexts, there are numerous instances of it landing short of even very simple commands [7-9]. More recently, ChatGPT went ‘viral’ on the internet for being unable to count the number of letter ‘R’s in the word ‘strawberry’ [10] - a task that seems very simple to most human individuals.

Several studies have explored the performance of ChatGPT across various medical examinations; in the United States Medical Licensing Examination (USMLE) [11], it demonstrated the equivalent of a passing score for a third-year medical student; in the Saudi Medical Licensing Exam (SMLE), it scored 88.6% [12], and it was able to pass the written tests for German medical state examinations [13]. There are numerous other success stories of LLMs (including ChatGPT) passing examinations, such as family medicine exams [14], thoracic surgery exams [15], and anatomy exams [16]. However, there is also plenty of research suggesting that the problems faced by LLMs (difficulty processing images, inability to communicate in real life) have meant failure in examinations, such as orthopaedic board exams [17,18], ophthalmology board exams [19], core cardiology exams [20], and radiology exams [21]. Whilst results have varied, ChatGPT and similar AI systems have shown promise when answering questions, perhaps not always to the ability required to pass, but in most to be competitive in passing. Given its potential to pass various medical examinations, there is growing interest in how ChatGPT could assist healthcare professionals in practice.

The Prescribing Safety Assessment (PSA) is the national assessment of prescribing ability in the United Kingdom (UK). For all physicians to be allowed prescribing rights upon graduation, this examination must be passed. Given the high rates of prescribing errors found in several large studies [22-24], the British Pharmacological Society (BPS) and the Medical Schools Council (MSC) Assessment collaborated to develop the PSA [25], which was officially implemented in 2014. The assessment itself is partially open-book, with medical students being allowed the use of the British National Formulary (BNF). The BNF is the UK’s pharmaceutical reference book, which is used by physicians across the country to aid in safe prescribing. The examination includes 60 questions and is two hours in length, with the majority of questions being multiple choice and some being free-text; results strictly pass or fail, and the pass mark is set using the Modified Angoff method, a standard-setting process that involves expert judgement to determine the minimum competence level required for safe prescribing. Students must pass the examination to obtain prescribing rights, and failure to do so requires re-examination until a passing score is achieved. The PSA assesses the ability to prescribe, review prescriptions, identify adverse drug reactions as well as drug interactions, and recognise prescribing errors. These broadly comprise the eight sections of the examination: prescribing, prescription review, planning management, providing information, calculation skills, adverse drug reactions, drug monitoring, and data interpretation. A wide variety of situations a junior physician may find themselves in are tested: medicine, surgery, elderly medicine, paediatrics, psychiatry, obstetrics and gynaecology, and general practice.

To the best of our knowledge, following a comprehensive review of the literature conducted on databases (PubMed, Scopus, Google Scholar, and Medline) using keywords such as 'ChatGPT', 'large language models', 'Prescribing Safety Assessment', and 'artificial intelligence', no studies published as of September 22, 2024 evaluate the performance of ChatGPT or any other LLM on the PSA. This study’s aim was to measure and critically assess ChatGPT’s performance on the PSA, with the broader goal of exploring its potential role in improving safe prescribing.

## Materials and methods

Study design

This study was a quantitative, observational analysis focused on evaluating ChatGPT’s (version GPT-4) performance on the PSA using percentage scores and pass/fail thresholds derived from practice papers available to medical students. No human participants or external reviewers were involved, as the primary focus was the AI system's autonomous ability to answer PSA questions. The AI's responses were graded against the official PSA mark schemes, which outline the correct answers and criteria for passing.

Materials

The materials used in this study included ChatGPT (version GPT-4), accessed via OpenAI's interface, which was used to answer the PSA questions.

PSA practice papers and their mark schemes, publicly available for medical students, were chosen to replicate the conditions of the actual examination. Each paper contains 30 questions (half the length of the full PSA examination), designed to be completed in 60 minutes with 100 available marks. Three of the four papers included both a mark scheme and a pass mark; the fourth paper had a mark scheme but no pass mark. No questions were excluded to maintain the authenticity of the practice examination; one question required the interpretation of a graph, and this question was included in the final study.

The BNF served as the primary reference for drug-related information, replicating the open-book conditions allowed during the PSA.

Procedure

Each of the four PSA practice papers was administered to ChatGPT in three independent trials, resulting in a total of 12 assessments. To ensure unbiased results, each trial was conducted in a separate chat session, preventing ChatGPT from carrying over information from previous attempts.

For each assessment, a standardised prompt was used: ‘You will be taking a practice assessment for the 'Prescribing Safety Assessment', which will assess knowledge, skills, and judgement related to prescribing and supervising the use of medicines in the healthcare environment. I do not need you to explain the reasoning or relevant medical knowledge for each one. I need you to simply provide the answer in the format asked of the question. The answers will be assessed based on the correct doses/routes/drugs in the BNF'. Once the session began, ChatGPT received no further prompts or instructions, and it responded to each question as if taking the PSA exam. Each individual question was entered into the chat function, an answer was produced by the software, and the next question was then entered.

After collecting ChatGPT’s responses, each answer was manually graded according to the official PSA mark scheme. The grades adhered strictly to PSA criteria, and no modifications were made to the process. ChatGPT’s percentage scores were then calculated and compared to the pass marks provided for each paper. Since justifications for answers are not part of the PSA exam, ChatGPT was not asked to provide reasoning for its responses.

Evaluation and metrics

ChatGPT’s performance was evaluated through two primary approaches: Overall Performance: The final percentage score for each practice paper was calculated by dividing the total number of correct answers by the total possible marks (100). This score was then compared against the predetermined pass mark for each PSA practice paper. This comparison allowed for an assessment of whether ChatGPT would have passed or failed the PSA based on its performance in the practice exams.

Section-wise analysis: To gain more detailed insights into ChatGPT’s abilities, its performance was also analysed on a section-by-section basis. The PSA is divided into eight sections: prescribing, prescription review, planning management, providing information, calculation skills, adverse drug reactions, drug monitoring, and data interpretation. For each section, the number of correct responses was calculated and compared to the total number of questions in that section. This analysis aimed to identify whether ChatGPT demonstrated particular strengths or weaknesses in specific areas. Performance trends across the three trials were also examined to assess consistency in each section.

Statistical analysis

For each PSA practice paper, the percentage scores from the three independent trials were averaged by calculating the mean to provide a representative score for that paper. This averaged score was compared to the official pass mark to assess whether ChatGPT would have passed the PSA based on its performance across multiple trials. Any variability between trials was also considered to evaluate the consistency of the AI’s performance. Variability was measured by the range.

In addition to the overall score, ChatGPT’s performance in each section of the practice papers was analysed to identify any areas of strength or weakness. For each section (e.g., prescribing, prescription review, calculation skills), the percentage of correct answers was calculated. Patterns or trends in performance across the three trials were examined to assess whether ChatGPT demonstrated consistency in certain areas or exhibited variability. Variability was measured by the range.

Ethical considerations

As this study did not involve human participants and utilised only publicly available materials, formal ethical approval was not required. Additionally, no privacy concerns or issues related to patient confidentiality were present, as all data were publicly accessible and scenarios given in the PSA practice papers were anonymised or devised by BPS.

## Results

Overall performance across practice papers

**Figure 1:**
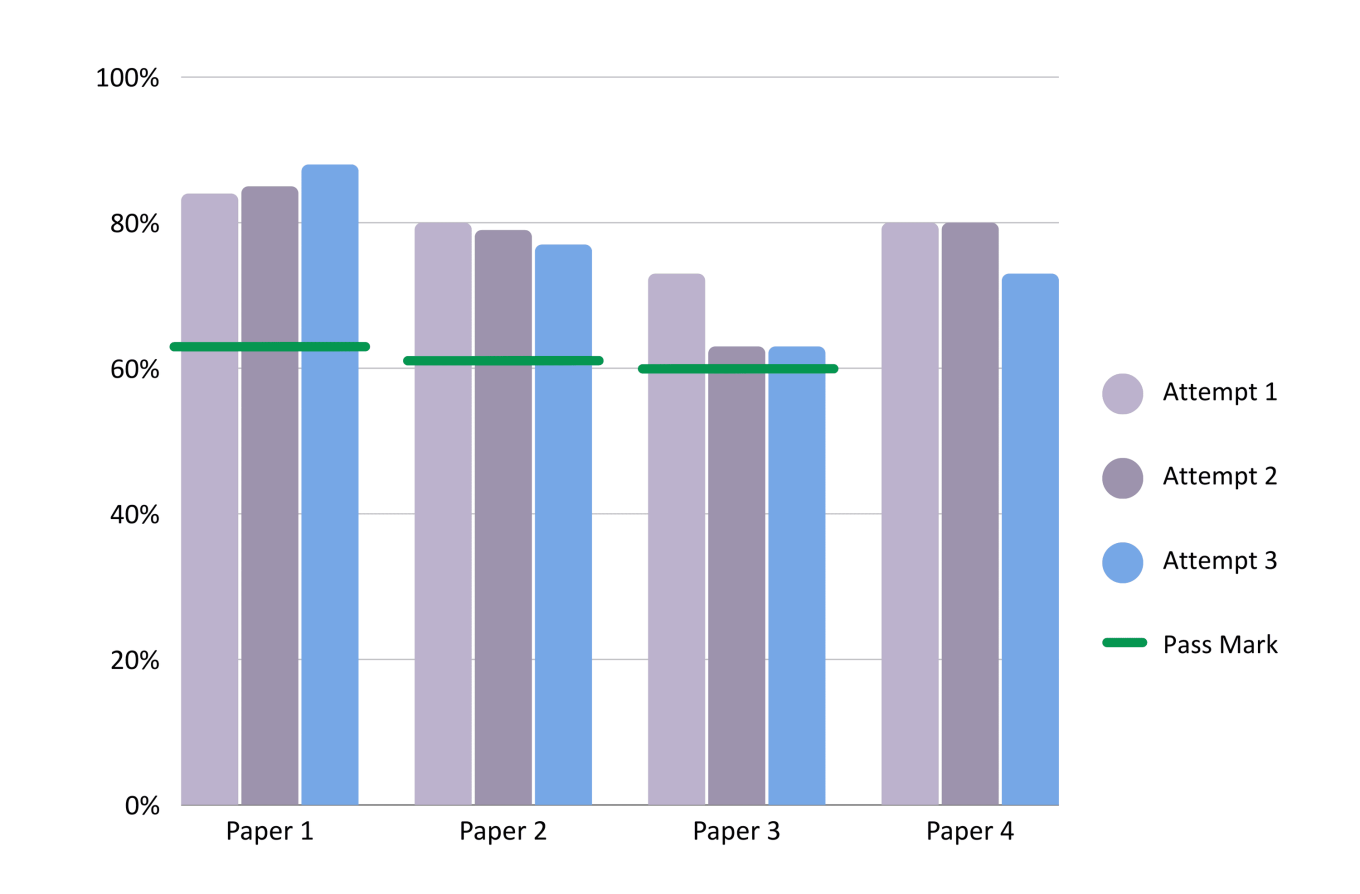
Performance of ChatGPT on the Prescribing Safety Assessment across different practice papers Percentage scores obtained by ChatGPT (version GPT-4) across four official practice papers of the Prescribing Safety Assessment. Each practice paper has three separate attempts. The official mark scheme was used to grade papers, with the pass mark pre-determined and displayed as a horizontal bar across each attempt of the paper. Paper 4 has no official pass mark. The Y-axis depicts the percentage score obtained; the X-axis depicts each attempt.

ChatGPT’s performance on the four PSA practice papers is summarised in Table 1. The AI achieved mean scores of 257/300 (85.67%), 236/300 (78.67%), 199/300 (66.33%), and 233/300 (77.67%) on papers 1, 2, 3, and 4, respectively. In papers 1, 2, and 3, ChatGPT’s scores surpassed the respective pass marks of 63.00%, 60.50%, and 60.00%. For paper 4, although no official pass mark was provided, ChatGPT achieved a mean score of 233/300 (77.67%), which aligned with its performance in previous papers, all of which surpassed the respective pass marks. Across all attempts, ChatGPT would have achieved a passing grade in all of the papers where a pass mark was available. Even in the trials without an official pass mark for paper 4, ChatGPT’s lowest score of 73/100 (73.00%) was 10.00% higher than the highest pass mark available based on paper 1. As shown in Figure 1, which depicts the scores across all four papers and all three attempts for each paper, ChatGPT consistently achieved scores above the pass marks.

**Table 1:** Performance of ChatGPT on the prescribing safety assessment across different practice papers Percentage scores obtained by ChatGPT (version GPT-4) across four official practice papers of the Prescribing Safety Assessment. Each practice paper has three separate attempts and the mean percentage displayed. The official mark scheme was used to grade papers, with the pass mark pre-determined and displayed.

Paper	Attempt 1/100 (%)	Attempt 2/100 (%)	Attempt 3/100 (%)	Mean/300 (%)	Pass mark (%)	Mean pass (yes/no)
1	84 (84.00)	85 (85.00)	88 (88.00)	257 (85.67)	63.00	Yes
2	80 (80.00)	79 (79.00)	77 (77.00)	236 (78.67)	60.50	Yes
3	73 (73.00)	63 (63.00)	63 (63.00)	199 (66.33)	60.00	Yes
4	80 (80.00)	80 (80.00)	73 (73.00)	233 (77.67)	N/A	N/A

Performance across different subsections

**Figure 2:**
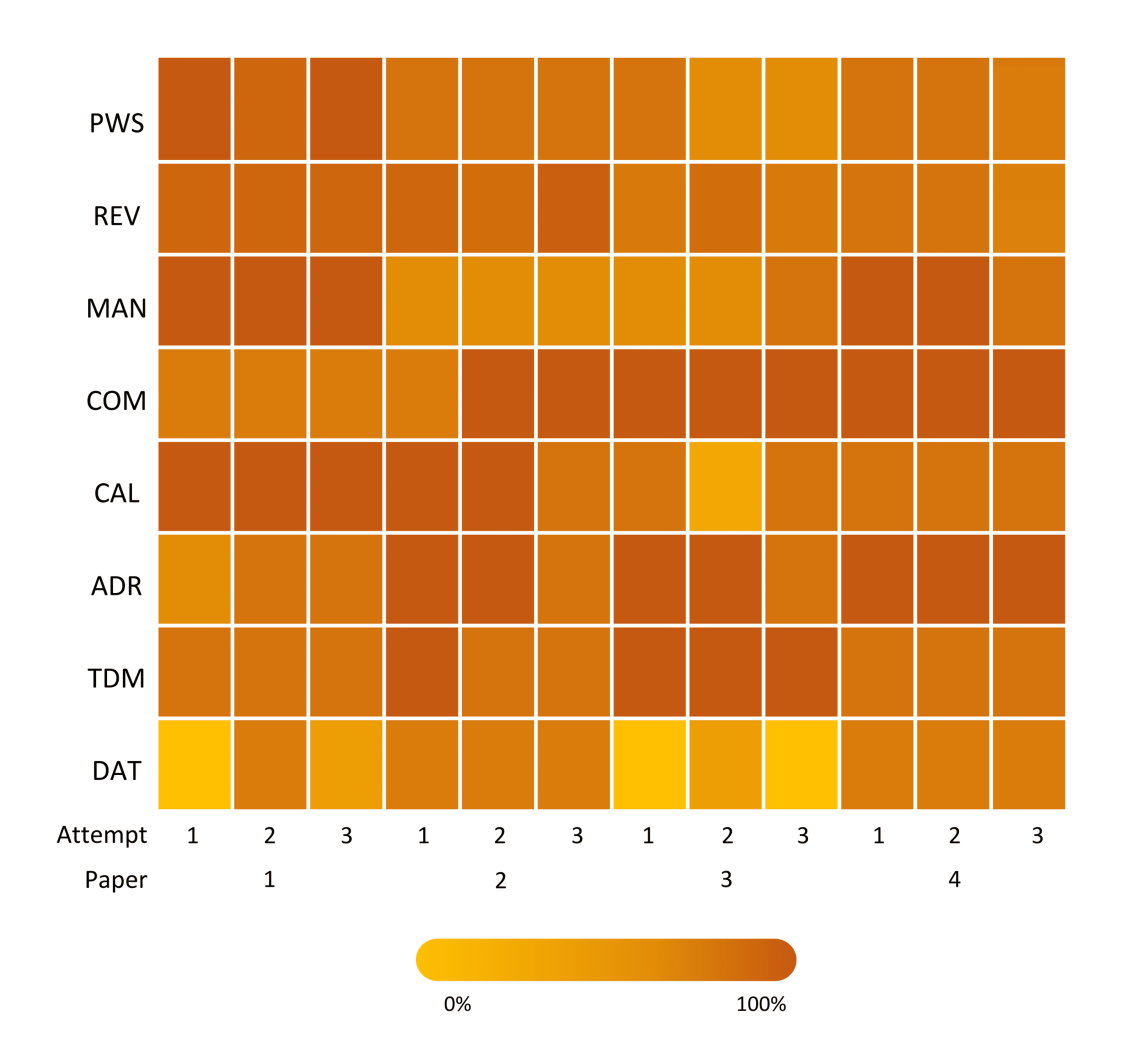
Performance of ChatGPT on the Prescribing Safety Assessment across different subsections Visual heatmap depiction of the percentage scores obtained by ChatGPT (version GPT-4) across eight subsections of the Prescribing Safety Assessment, across four official practice papers. Each practice paper has three separate attempts. The Y-axis depicts each subsection of the examination, the X-axis depicts each attempt. PWS: prescribing; REV: prescription review; MAN: planning management; COM: providing information; CAL: calculation skills; ADR: adverse drug reaction; TDM: drug monitoring; DAT: data interpretation.

ChatGPT’s performance across individual subsections of the PSA is presented in Table 2. The AI demonstrated variable performance across the eight subsections, with the highest mean scores in 'Communicating Information', scoring 64/72 (88.89%), and 'Adverse Drug Reaction', scoring 63/72 (87.50%). Conversely, the AI underperformed in 'Data Interpretation', scoring 32/72 (44.44%). A wide range of results was observed within individual subsections, particularly in 'Planning Management', where ChatGPT scored 24/24 (100.00%) in Paper 1, but only 12/24 (50.00%) in Paper 2. Similarly, 'Data Interpretation' demonstrated considerable variability, with scores ranging from 12/18 (66.67%) to as low as 2/18 (11.11%). A heatmap of the results, displayed in Figure 2, illustrates ChatGPT’s performance across all subsections of the four papers in all attempts. The lowest percentage scored in a trial was 0/18 (0.00%) in data interpretation, whilst the highest was 100.00% in all other subsections except prescription review.

**Table 2:** Performance of ChatGPT on the Prescribing Safety Assessment across different subsections Percentage scores obtained by ChatGPT (version GPT-4) across eight subsections of the Prescribing Safety Assessment. Performance is broken down by four different practice papers, with a total average percentage score calculated for each subsection. Each practice paper has three separate attempts contributing to an average final percentage displayed in the table.

Subsection	Paper 1 n (%)	Paper 2 n (%)	Paper 3 n (%)	Paper 4 n (%)	Total n (%)
Prescribing	115/120 (95.83)	105/120 (75.00)	70/120 (58.33)	87/120 (72.50)	362/480 (75.42)
Prescription Review	42/48 (87.50)	42/48 (87.50)	35/48 (72.92)	34/48 (70.83)	153/192 (79.69)
Planning Management	24/24 (100.00)	12/24 (50.00)	14/24 (58.33)	22/24 (91.67)	72/96 (75.00)
Communicating Information	12/18 (66.67)	16/18 (88.89)	18/18 (100.00)	18/18 (100.00)	64/72 (88.89)
Calculation Skills	24/24 (100.00)	22/24 (91.67)	14/24 (58.33)	18/24 (75.00)	78/96 (81.25)
Adverse Drug Reaction	16/24 (66.67)	22/24 (91.67)	22/24 (91.67)	24/24 (100.00)	84/96 (87.50)
Drug Monitoring	18/24 (75.00)	20/24 (83.33)	24/24 (100.00)	18/24 (75.00)	80/96 (83.33)
Data Interpretation	6/18 (33.33)	12/18 (66.67)	2/18 (11.11)	12/18 (66.67)	32/72 (44.44)

Consistency across multiple attempts

To assess the consistency of ChatGPT’s performance, each practice paper was repeated three times. The overall mean scores remained relatively stable across attempts, with minor fluctuations. Paper 1 ranged from 84/100 (84.00%) to 88/100 (88.00%), and paper 2 ranged from 77/100 (77.00%) to 80/100 (80.00%). However, more pronounced differences were observed in papers 3 and 4, where scores ranged from 63/100 (63.00%) to 73/100 (73.00%) for paper 3, and 73/100 (73.00%) to 80/100 (80.00%) for paper 4. Figure 1 displays ChatGPT’s performance across multiple attempts for each paper, visually highlighting the relatively stable results in papers 1 and 2, as well as the more significant fluctuations in papers 3 and 4.

## Discussion

The results of this study demonstrate ChatGPT’s strong performance in the PSA, surpassing the pass mark across all practice papers (where a pass mark was available). This reinforces findings from previous studies on medical examinations, such as the USMLE [11] and SMLE [12], which also showed that AI models could compete with human medical students in certain standardised assessments. However, the variability in ChatGPT’s performance across different sections of the PSA underscores important limitations that must be considered. These limitations must be addressed before the widespread use of AI in prescribing assistance and medical training. Structured information given to AI can help it learn better, thus making its future outputs more effective. By utilising AI’s strengths, this can aid prescribers, especially in areas where high volumes of factual recall would be needed to identify all possible interactions and potential adverse drug reactions.

Equivalence to a legal prescriber

ChatGPT’s ability to pass the PSA raises important questions regarding its potential role as a legal prescriber. In the UK, passing the PSA is a fundamental requirement for junior physicians to be granted prescribing rights. However, while its performance suggests it meets the technical threshold for safe prescribing, the concept of equating an AI system to a legal prescriber is far more complex.

The legal and ethical considerations must be addressed. Current regulations do not recognise AI models as independent entities capable of assuming clinical responsibilities. A physician’s role extends beyond prescribing medications; it involves clinical judgement to weigh individual factors, engage in multidisciplinary collaboration, and communicate with patients. These dynamic variables are areas where AI currently lacks the capacity to perform independently, reinforcing the need for physician oversight. Accountability in error is another critical issue. In the event of a prescribing error, who bears the responsibility? The developer of the AI model, the healthcare institution, or does ChatGPT need a clinical supervisor like all junior physicians?

Whilst ChatGPT demonstrated competence in the PSA, this does not account for its ability to manage real-time changes in patient conditions, incomplete medical histories, or unexpected drug reactions. Human oversight would still be necessary to ensure safe prescribing.

Perhaps the largest issue is, of course, that passing one examination does not make ChatGPT a physician. It is the combination of passing multiple examinations and being signed off on clinical attachments and various formats of examinations (such as face-to-face clinical examinations) that are required to be a physician in the UK. ChatGPT has shown it can pass one of them in this study, and some other studies suggest it could pass others [13-16,26], but there is a long way to go before it can be considered independent.

Variable sectional performance

ChatGPT excelled in sections such as 'Communicating Information' and 'Adverse Drug Reaction'. The latter subsection of the examination tests students’ knowledge of the type of adverse event a drug may unfortunately exhibit, interactions between medications that could lead to unwanted events, or management of reactions to drugs; these questions, which are based on factual recall, are typically areas where one would think AI could have a potential role in aiding safe prescribing. A perhaps surprising result would be where communication comes in; ChatGPT showed very strong results for what information should be given to patients when starting a new medication, what should be communicated when stopping contraceptives, and when patients should expect to be monitored and how (blood tests, BMI measurement, etc.). This type of information, although assessed as part of a question type which requires communication, a particularly human skill, is really testing, again, factual recall rather than the student’s ability to communicate with a patient, it is testing the knowledge needed in order to communicate. The ability to communicate with patients is a skill commonly assessed in face-to-face clinical examinations. It forms higher-level aspects of being a physician, over and above factual recall. Again, having an AI model perform as an independent prescriber would fall short of the integral aspects of human interaction and physical communication required in most prescribing scenarios.

The section where AI clearly struggled the most was in 'Data Interpretation'. This section requires a critical analysis of data and an understanding of what changes need to be made to improve upon already existing data points. This is in stark contrast to the rest of the study, where factual recall is most important; this section tests the ability to think critically and, in some questions, requires no knowledge whatsoever, just the ability to use excellent logic and ability to respond appropriately to any changes in clinical status. This limitation of AI in the examination aligns with existing literature, which has identified areas such as image-based diagnosis or data processing as weak points in current AI systems [27,28], although changes are being made quickly to improve this [29]. This dichotomy between strong performance in fact-recall sections and underperformance in areas requiring critical thinking reflects a common limitation in LLMs. AI models like ChatGPT are designed to retrieve and present information from vast databases but struggle when required to interpret ambiguous or incomplete data, as is often the case in clinical settings.

Implications for AI-assisted prescribing

More junior physicians are prone to prescribing errors [22-24], particularly in the early years of practice. AI tools could help play a role in reducing errors by offering real-time assistance, especially in areas such as adverse drug interactions or dose calculations. Both of these areas are places where ChatGPT scored highly in the PSA. Some electronic prescribing systems are in use in the UK and have basic tools to help identify specific common errors. An anecdotal example is the use of Cerner PowerChart in many London hospitals. If a physician attempts to prescribe a penicillin antibiotic to a patient with a registered penicillin allergy, an alert pops up on the screen to prevent (or attempt to prevent) the prescription from being signed. This relies on the allergy being recorded, and some Cerner versions do not have this feature. Thus, there remains room for error; however, with more specialised AI tools, the error rate could perhaps be improved. Providing structured data inputs to AI models, such as strict adherence to the BNF or similar resources, could further enhance their utility by making their outputs more reliable and focused. This would allow AI to better assist junior physicians in reducing errors, especially in environments where prescribing decisions must be made quickly and accurately.

Comparison to human performance

Human students can reason, adapt to unexpected circumstances, and consult resources dynamically. While ChatGPT can retrieve information very quickly and efficiently, the nuance of human judgement and the ability to interpret a patient’s unique situation remain vitally important. Low scores in 'Data Interpretation', and occasional low scores across the board in other subsections such as “Planning Management” show its limitations in clinical decision-making, where contextual factors can greatly influence decisions. In its current form, AI seems well suited to supplementing rather than replacing human decision-making in healthcare and prescribing.

Limitations of this study

Whilst the study provides valuable insights into ChatGPT’s performance on the PSA, several limitations must be acknowledged, particularly when determining if ChatGPT is safe as an independent prescriber.

Practice Papers Only

The study was conducted using only practice papers available online. Although these papers are designed to reflect the actual PSA format and are created/written by the same PSA writers, they may not fully encompass the complexity or breadth of questions seen in the real assessment. Additionally, the total number of practice papers was limited, which may constrain the scope of the findings. A more comprehensive evaluation using a larger set of PSA and PSA-like questions could provide a more accurate reflection of the AI’s prescribing safety.

Access to Correct Sources

Although prompted to use the BNF as its primary data source, it is very difficult to tell where ChatGPT received its information from. In a real exam setting, students have access to the BNF only, whereas (although instructed to only use the BNF) ChatGPT, of course, was able to use the full breadth of the internet at its disposal. Although this helps us understand whether ChatGPT is helpful in safe prescribing (after all, in real life, one is not limited to just the BNF), it does make it harder to tell whether (using only the BNF) ChatGPT would have passed the PSA.

Not Real-World

This study’s primary aim was to determine if ChatGPT would pass the PSA, and using practice questions only proves it can and likely would. However, the secondary aim of evaluating its benefit in the real world is much harder, as ChatGPT was evaluated on pre-set questions rather than real clinical scenarios. AI models may struggle with the nuances of real-life clinical situations, such as patient-specific variables, incomplete data, or the need for interdisciplinary consultation. This lack of real-world application in the testing environment restricts the extrapolation of the results found in this study.

Reasoning Process

This study did not qualitatively analyse ChatGPT's reasoning process. This is not a requirement in the PSA but would be crucial for real-world prescribing. Physicians must be able to explain their reasoning and justify their prescriptions. ChatGPT’s answers were graded on accuracy to the mark scheme only and may have received points for correct answers where it did not truly understand. Of course, the same can be said for students too! However, the question of whether MCQ-style questions are best to test students has been debated vigorously [30-32] and goes beyond the remit of this study.

Version GPT-4

Although not discussed at length here, ChatGPT version GPT-4 was used - the latest version of ChatGPT at the time of writing. Future iterations of AI models may differ significantly, and comparisons between versions GPT-3.5 and GPT-4 have shown measurable differences, with GTP-4 being far improved [33-35]. Continuous updates to the model and its training data could enhance future performance. Of course, other AI models are available; at the time of writing, Gemini (Google’s AI chatbot) was starting to become popular and had recently been tested on medical examinations [19]. The results of this study therefore are limited in generalisability to newer ChatGPT models and other AI platforms. AI models are constantly being refined, and the limitations observed in this study could be addressed in future versions, where newer models could integrate more sophisticated reasoning algorithms or gain improved access to structured medical databases, further reducing variability and enhancing their reliability in clinical decision-making.

Future work

A focus on expanding the scope of AI evaluation within medical examinations is important, and this study places itself amongst the many others on testing ChatGPT’s ability within specific examinations. It must be said that ChatGPT is built to use all the knowledge available on the internet, can be used for a huge variety of tasks [36-40], and is not specifically built to be used within medicine. Implementation of AI into electronic medical records should be trialled, specifically within the boundaries of where AI currently performs well (adverse drug reactions and communicating information in a prescribing setting), and by enhancing the AI software and developing it for specific requirements, as well as providing it specific sources and information to use, akin to Mycin [2,3], the AI can learn from previous users decisions [41] and knowledge in a safe, secure system, allowing future users to have better informed decisions. By implementing structured learning systems and better refining the sources AI can draw from, future models could provide more accurate and contextually relevant recommendations. This would allow AI tools to become more adept at handling clinical decisions in real-time, especially as they evolve based on user feedback and real-world data. The PSA was specifically introduced to aim to reduce junior physicians’ prescribing errors; longitudinal studies could examine whether integrated AI tools result in a measurable reduction in prescribing errors over time.

The use of AI in healthcare is likely to grow, particularly in areas like prescribing where errors can lead to significant patient harm. However, integration must be approached with caution, especially with a large number of UK physicians now turning to ChatGPT for aid in writing medical letters and other administrative tasks, with some even using the software to suggest differential diagnoses [42]. Future iterations of AI must prioritise the ability to reason contextually and interpret clinical data in real-time. The goal should be to create a symbiotic relationship where physicians can guide AI to improve and AI can aid physicians with decisions; this will leverage the strengths of both to improve patient care.

## Conclusions

ChatGPT demonstrated strong potential in performing tasks related to safe prescribing, passing the PSA examination based on its responses to multiple practice papers. The AI’s performance in sections such as adverse drug reactions and drug monitoring suggests that it could assist in reducing prescribing errors, especially among junior physicians. However, the variability in its performance, particularly in data interpretation, indicates that AI is not (yet) a perfect substitute for human judgement in clinical practice. AI systems similar to ChatGPT should be seen as tools that can augment the skills and judgement of healthcare professionals, helping to mitigate common prescribing errors rather than replacing human oversight and critical thinking.
